# Changes in Weight and Nutritional Habits in Adults with Obesity during the “Lockdown” Period Caused by the COVID-19 Virus Emergency

**DOI:** 10.3390/nu12072016

**Published:** 2020-07-07

**Authors:** Marianna Pellegrini, Valentina Ponzo, Rosalba Rosato, Elena Scumaci, Ilaria Goitre, Andrea Benso, Sara Belcastro, Chiara Crespi, Franco De Michieli, Ezio Ghigo, Fabio Broglio, Simona Bo

**Affiliations:** 1 Department of Medical Sciences, University of Torino, c.so AM Dogliotti 14, 10126 Torino, Italy; marianna.pellegrini@unito.it (M.P.); elena.scumaci@libero.it (E.S.); ilaria.goitre@libero.it (I.G.); andrea.benso@unito.it (A.B.); sara.belcastro@unito.it (S.B.); chiaracrespi@hotmail.it (C.C.); franco.demichieli@unito.it (F.D.M.); ezio.ghigo@unito.it (E.G.); fabio.broglio@unito.it (F.B.); 2 Department of Psychology, University of Torino, c.so AM Dogliotti 14, 10126 Torino, Italy; rosalba.rosato@unito.it; 3 Diabetes and Metabolic Diseases Clinic, “Città della Salute e della Scienza” Hospital of Torino, 10126 Torino, Italy

**Keywords:** COVID-19 infection, dietary habits, lockdown, obesity

## Abstract

Our aim is evaluating the changes in weight and dietary habits in a sample of outpatients with obesity after 1 month of enforced lockdown during the COVID-19 pandemic in Northern Italy. In this observational retrospective study, the patients of our Obesity Unit were invited to answer to a 12-question multiple-choice questionnaire relative to weight changes, working activity, exercise, dietary habits, and conditions potentially impacting on nutritional choices. A multivariate regression analysis was performed to evaluate the associations among weight/BMI changes and the analyzed variables. A total of 150 subjects (91.5%) completed the questionnaire. Mean self-reported weight gain was ≈1.5 kg (*p* < 0.001). Lower exercise, self-reported boredom/solitude, anxiety/depression, enhanced eating, consumption of snacks, unhealthy foods, cereals, and sweets were correlated with a significantly higher weight gain. Multiple regression analyses showed that increased education (inversely, β = −1.15; 95%CI −2.13, −0.17, *p* = 0.022), self-reported anxiety/depression (β = 1.61; 0.53, 2.69, *p* = 0.004), and not consuming healthy foods (β = 1.48; 0.19, 2.77, *p* = 0.026) were significantly associated with increased weight gain. The estimated direct effect of self-reported anxiety/depression on weight was 2.07 kg (1.07, 3.07, *p* < 0.001). Individuals with obesity significantly gained weight 1 month after the beginning of the quarantine. The adverse mental burden linked to the COVID-19 pandemic was greatly associated with increased weight gain.

## 1. Introduction

The pandemic of the coronavirus SARS-CoV-2 (COVID-19) has caused significant disruption in everyday lifestyle. In Italy, at the beginning of March, an important growth in infections and deaths was observed [1]; the whole country became a protected zone, with severe restrictive national measures, such as the closure of all activities not considered essential, including schools/university, sport activities, shops, and factories [2,3]. People had to stay at home and were only allowed to go out to buy food or for health reasons; all working activities were suspended or turned into smart working at home, except for essential activities (health workers, food supply and sale, cleaning of cities, and police, etc.).

Therefore, since the 10th of March, 2020, the month of the start of the “lockdown”, millions of Italians were forced to remain at home. This enforced quarantine can have a heavy psychological impact, above all among persons with obesity who are already at risk of social isolation and experiencing higher rates of depression [4]. The mental health burden during the COVID-19 outbreak has been evaluated by a few studies, and an increased rate of anxiety disorder, depressive symptoms, perceived stress, post-traumatic stress disorder, and poor sleep quality has been reported [5,6,7,8,9,10]. Usual lifestyle habits have been heavily disrupted by the mandatory stay-at-home orders, which may result in important behavior changes, particularly dietary habits, in this kind of natural experiment, “forced” by an unpredictable emergency [11]. The rise in unstructured time might induce overeating and increase screen time. Furthermore, social isolation might worse lifestyle behaviors with enhanced sedentarism, as well as decreased outdoor time and increased weight gain [12]. It has been hypothesized that the increased out-of-school time may exacerbate the weight gain of children in a similar way to summer recess [13]. No other data about the effects of quarantine in adult individuals with obesity are currently available.

The objective of the present observational study was to evaluate the changes in weight and dietary habits in a sample of individuals with obesity attending our Obesity Unit after 1 month of enforced lockdown.

## 2. Materials and Methods

This was an observational retrospective study. All the patients of the Obesity Unit of the Diabetes and Metabolic Diseases Clinic of the Città della Salute e della Scienza Hospital of Torino were enrolled. We included in the study all the patients who currently attend the weight loss program. In our unit, we take care of patients with BMI >30 kg/m^2^ and <45 kg/m^2^, aged 18–75 years. Dropouts (i.e., those who do not attend the scheduled appointment and either do not answer a call or declare that they no longer want to participate in the program) were excluded from the study.

### 2.1. Weight Loss Program

Our patients followed a 12-month multidisciplinary weight loss program that included at least six meetings. Supplementary visits could be scheduled in specific cases, such as the prescription of drugs or a very-low-calorie diet. During the first appointment, the patients received two group sessions (one by trained nurses who welcomed participants, evaluated the motivation, and discussed their strengths, weaknesses, opportunities, and one of nutritional education by dieticians) and individual visits with the endocrinologists, the psychologists and the dieticians. During follow-up, participants were initially visited by dieticians and endocrinologists every month and, after the first 90 days, every 3 months, with supplementary visits in case of specific needs.

All patients received verbal and written dietary, exercise, and behavior recommendations. A personalized diet was prescribed according to the Mediterranean diet (45–55% carbohydrates, <10% sugars, 30% fats, <10% saturated fats, 15–25% proteins, and 20–30 g fiber) with a 500–1000 kcal energy restriction, based on the individual usual assumption and caloric needs. Patients were asked to weigh themselves every day.

A moderate exercise, such as at least 150 min/week of brisk walking plus 30 min/week of exercise against resistance, was prescribed and a leaflet on examples of home exercises was given to all patients. Psychologists specialized in obesity treatment screened all attenders during the first visit to evaluate the need for further individual sessions for selected patients during the weight-loss program; a group session about motivation, how to recognize and manage emotional eating, relapse prevention, and self-manage social gatherings was delivered by the psychologists during the second visit.

### 2.2. Questionnaire

The research team developed a series of items which were then grouped and prioritized, based on a review of the literature, the feedbacks obtained during our group sessions about patients’ difficulties in diet maintenance, and a consultation with clinical experts. A 12-question multiple-choice questionnaire was prepared. A cognitive debriefing of the questionnaire was performed with 10 patients selected using the method of purposive sampling. Interview content was analyzed informally. After providing informed consent, patients completed the questionnaire; a psychologist (ES), experienced at interviewing, conducted individual telephone interviews with patients with the aim to assess the readability of the questions and the acceptability of the questionnaire. Two questions, which were deemed unclear, were then reformulated.

The questions were relative to the weight before and after 1 month of lockdown and working activity and exercise during quarantine, with many items related to the changes in dietary habits and the conditions potentially impacting on nutritional choices. Exercise during the lockdown was considered as practicing at home (indoor exercise bike, treadmill, and resistance training) or jogging/running around the house, given that it was not possible either to participate in organized sports or perform other outdoor activities. A snack was considered as every eating occasion between main meals. According to our weight loss program, patients should weigh themselves every day with the same scale. They were asked to report their weight on the 11th of March and at the date of the questionnaire compilation (see below). Night eating syndrome was diagnosed in the presence of recurrent episodes of night eating, as manifested by eating after awakening from sleep or by excessive food consumption after the evening meal, with awareness and recall of the eating, without association with effects of medications, another medical or mental disorder, or external influences (such as changes in the individual′s sleep–wake cycle or local social norms), in line with the DSM-5 criteria [14]. Weight cycling was defined in the presence of >2 weight-loss cycles followed by weight regain.

A dedicated researcher contacted the patients by phone and explained to the patients how to fill out the questionnaire. The questionnaire was sent by e-mail on the 14th of April, 2020. The patients completed it in autonomy and returned it between the 14th of April and the 21st of April. Patients were strongly requested to be truthful and to answer carefully in order to ensure the quality of the survey. In 15 cases, patients were unable to fill in the questionnaire by email and completed it by telephone interview with the same researcher.

Clinical and sociodemographic data referring to the first ambulatory visit were extracted from the clinical records. At the first visit, the following anthropometric measurements were assessed: weight (measured with the participant wearing light clothes and no shoes to the nearest 0.1 kg by a mechanical column scale, SECA model 711, Hamburg, Germany), height (measured to the nearest 0.1 cm with a Stadiometer, SECA 220 measuring rod, Hamburg, Germany), waist and neck circumferences (measured by a plastic tape meter respectively at the umbilicus level or under the cricoid cartilage).

### 2.3. Ethical Aspects

Each patient gave his/her verbal informed consent to participate to the study. The study protocol was approved by the Ethics Committee of the Città della Salute e della Scienza Hospital of Turin.

### 2.4. Statistical Analyses

Within-group weight and BMI variations were analyzed by *t*-test for paired samples. The changes in weight and BMI (values after 1-month of lockdown minus values before quarantine) did not show a normal distribution. Differences in weight/BMI changes were analyzed by Mann–Whitney test (two groups) or by Kruskal–Wallis test (three groups). A multiple regression model was performed to evaluate the association among weight/BMI changes and the variables which resulted significantly different between the various categories. A regression path model was applied to test the hypothesis that not healthy food choice could mediate the effects of self-perceived anxiety/depression on weight gain.

## 3. Results

A total of 164 patients were invited to participate in the survey; 150 participants completed the questionnaires and were included in the analysis, while 14 (8.5%) either refused to participate, were not reachable, or provided incomplete answers to the questionnaire. The baseline characteristics of the participants are described in Table 1 and did not significantly differ from those of non-participants (data not shown). The mean duration of the attendance at our Obesity Unit was 6.7 months (Table 1).

On average, during the lockdown period, self-reported weight and BMI significantly increased by 1.51 kg (*p* < 0.001 by *t*-test for paired samples) and 0.58 kg/m^2^ (*p* < 0.001), respectively.

The answers to the questionnaire are shown in Table 2. Most individuals either did not exercise or reduced their level of physical activity, and a great number of unhealthy nutritional behaviors were evident.

The changes in weight and BMI according to those answers are shown in Table 3. A significantly higher weight and BMI increase was evident in individuals with lower education, who reported lower exercise, self-reported boredom/solitude, anxiety/depression, enhanced eating, and consumption of unhealthy foods, snacks, cereals, and sweets.

The increased consumption of snacks, cereals, and sweets were all highly correlated (*p* < 0.001 by the chi-square test). By assessing their individual association with both weight and BMI changes in a multiple regression model, after adding education and exercise level, self-reported boredom/solitude, and anxiety/depression, only sweets consumption was significantly associated with weight/BMI changes. Then, a multiple regression analysis was run, with the inclusion of all the variables significantly associated with increased weight and BMI, except cereals and snacks consumption (Table 4). No multicollinearity between variables included in the multivariate analysis was found, since all variables showed a variance inflation factor lower than 1.3.

Lower education level, self-reported anxiety/depression, and not consuming healthy foods were significantly associated with increased weight and BMI gain during the lockdown period.

To evaluate whether not-healthy food choices could mediate the effects of self-reported anxiety/depression on weight gain, a regression path model was applied. In the presence of self-reported anxiety/depression, the estimated weight gain was 2.69 kg (95%CI 1.66–3.71; *p* < 0.001); the estimated direct effect of anxiety/depression was 2.07 kg (95%CI 1.07–3.07; *p* < 0.001), while the indirect effect linked to unhealthy choices accounted for 0.62 kg (95%CI 0.16–1.08; *p* = 0.008).

## 4. Discussion

We found that patients with obesity attending an Obesity Unit in Northern Italy showed a ≈1.5 kg self-reported weight gain after the first month of lockdown. Lower education level, self-reported anxiety/depression, and not paying attention to the healthiness of food choices were significantly associated with weight and BMI increase.

Italy was the first European country to be deeply affected by the Covid−19 pandemic and to undertake severe restrictive measures at a national level. This implied a greater risk of sedentarism and exercise reduction on one side, but on the other, an increased possibility to eat almost every meal at home, the impossibility to consume restaurant/cafeteria meals, and the availability of more time to cook, with lower need for ready meals, usually rich in fats, sugars, and salt. Accordingly, most of our patients showed a reduction both in their exercise level and, likely, in physical activity during work, as only 15% continued to go to the workplace during quarantine. Indeed, even though all our patients received personalized nutritional advice and on average have been attending our unit for six months, they reported many unhealthy dietary habits, such eating more (40%), not paying attention to the healthiness of the consumed food (28%), consuming more sweets (50%), more snacks (33%), more frozen/canned foods (17%), and less fruit and vegetables than before (18%). Among those, not paying attention to the healthiness of food was the behavior most strongly associated with weight gain, with a more than 50% increased risk. In Italian children and adolescents with obesity, unfavorable changes in eating, sleep, and activity behaviors occurred during the lockdown period with the school closure [15]. No data about their weight changes were available. Thus, these were the first available data about changes in nutritional habits and weight during the current quarantine.

Low education might be a proxy for low socioeconomic level, a condition potentially impacting on food choice (greater purchase of more shelf-stable, highly processed foods at the expense of less processed, less energy-dense, fresh, perishable foods which are usually more expensive). Indeed, the increased social isolation, loneliness, boredom, anxiety, and depression generated by the pandemic might have played major roles in the lifestyle changes. In particular, self-reported anxiety/depression was the strongest predictor of weight gain in our patients. It is well known that emotional changes and mood disorders influence food choices, with the search for comfort foods, such as processed snacks and sweets [16,17,18]. An increased rate of depression and anxiety disorders has been described during the COVID-19 pandemic [5,6,7,8,9,10]. The anxiety/depression reported by our patients was strongly associated with weight gain and resulted in being the more relevant factor in predicting increase in body weight, after adjusting for consuming unhealthy foods.

These data suggest the pressing need to provide individuals with obesity—particularly those more vulnerable individuals—with tools, such as telemedicine instruments, to offer lifestyle information and interventions, and psychological support and guidance to maintain healthy choices, also in consideration of the uncertainty of the quarantine duration.

Furthermore, individuals with obesity are at increased risk of either chronic or acute diseases, including COVID-19 infection and complications, as suggested by growing evidence [19,20,21]. The increased risk is due to multiple factors [21]; in particular, excess ectopic fat might reduce both protective cardiorespiratory reserves, as well as potentiate the immune dysregulation and pro-inflammatory response, and have detrimental effects on lung function [22,23]. Finally, the consumption of unhealthy diets has been proposed to adversely impact on susceptibility to COVID-19 and recovery [24,25].

Increasing weight might be a vicious circle leading to increased infection risk so that, now, obesity and COVID-19 infection can be considered two public healthy pandemics colliding [4].

The present study had several limitations. The retrospective design of the study allowed us to estimate associations only and the sample size was small. Weight was self-reported and not measured; similarly, self-reported questionnaires were used. Those data may have been affected by bias and low reliability. Ad-hoc psychometric questionnaires were not employed. Anxiety and depression were combined in the same item. Other potential confounders have not been examined. The majority of our patients were women; however, the same gender distribution was reported by most published studies on outpatients as well [26,27,28].

Indeed, our results were consistent; our sample size (*n* = 150) achieved a 91% power with a significance level (alpha) of 0.05 to evaluate the associations with weight changes. This was the first study analyzing weight and dietary changes in adults during the lockdown period.

## 5. Conclusions

A small cohort of individuals with obesity significantly gained weight 1 month after the beginning of the lockdown period. The adverse mental burden linked to the COVID-19 pandemic might be associated with their increased weight. Further larger studies on this topic are needed to confirm these preliminary results obtained in a limited number of patients.

## Figures and Tables

**Table 1:** Clinical characteristics of participants before and after the lockdown period.

	Mean or %	SD
Age (years)	47.9	16.0
Males (%)	22.7	
Living alone (%)	49.3	
Not working/retired (%) *	40.7	
Active smokers (%)	16.0	
Alcohol consumption:		
Abstainers (%)	88.7	
<30 g/day alcohol (%)	11.3	
Secondary schools/graduation (%)	62.0	
Weight cycling (%)	53.3	
Night eating (%)	12.0	
Anthropometric values at the first ambulatory visit		
Weight (kg)	97.8	16.3
BMI (kg/m^2^)	36.6	4.5
Waist circumference (cm)	113.8	11.8
Neck circumference (cm)	37.4	3.7
Before the lockdown-period **		
Weight (kg)	92.0	17.0
BMI (kg/m^2^)	34.4	4.9
Months of outpatient follow-up	6.7	3.2
After the lockdown-period **		
Weight (kg)	93.5	17.5
Delta weight (kg)	+1.51 (−7.0 + 10.0) ^§^	
BMI (kg/m^2^)	35.0	5.2
Delta BMI (kg/m^2^)	+0.58 (−2.72 + 4.36) ^§^	

* this category included: 21.3% unemployed; 36.1% housewives; 37.7% retired; 4.9% students. ** self-reported weight; data collected by questionnaire. ^§^ (range).

**Table 2:** Answers to the Questions of the Questionnaire.

%	Answers	Question	%	Answers	Question
7.3	Yes, I habitually used those services even before the quarantine	During the lockdown period, have you used food delivery services?	33.3	Home working	**My current working situation is:**
6	Yes, I started using them during the quarantine		15.3	I still go to workplace	
8.7	I didn’t use them during quarantine, but I used them before		16.7	I haven’t been working since the beginning of the quarantine	
78	No, I did use them neither during quarantine, nor before		34.7	Unemployed/retired before quarantine	
28	I don’t consume snacks usually	During the lockdown period, the number of snacks that you consume in a day:	32.6	I never practice exercise	**During the quarantine period, the exercise that you have practiced:**
11.3	is less than before quarantine		46.7	is less than before quarantine	
28	is the same as before quarantine		10	is the same as before quarantine	
32.7	is more than before quarantine		10.7	is more than before quarantine	
2.7	I don’t consume those foods usually	During the lockdown period, your consumption of cereals (pasta, rice, other.):	13.3	No, I have maintained my eating habits	**Have you changed eating habits during the lockdown period?**
16	is less than before quarantine		34	Not too much, with a few exceptions	
53.3	is the same as before quarantine		40	I eat more than before quarantine	
28	is more than before quarantine		12.7	I eat less than before quarantine	
0.7	I don’t consume those foods usually	During the lockdown period, your consumption of sources of protein (meat, fish, eggs, cheese, legumes):	32.7	More time for cooking	**Which of the following conditions mainly impact on your eating habits? (you can choose more than 1 option)**
18	is less than before quarantine		21.3	Greater familial conviviality caused by forced domestic cohabitation	
54	is the same as before quarantine		36	Boredom/solitude	
27.3	is more than before quarantine		34.7	Anxiety/depression	
0.7	I don’t consume those foods usually		19.3	Continuous availability of food	
18	is less than before quarantine	During the lockdown period, your consumption of fruit and vegetables:	16	I consume/prepare more healthy foods, paying attention to the seasoning	**During the lockdown period, the healthy foods that you prepare/consume:**
54	is the same as before quarantine		56	I have not changed habits with respect to the type of food	
27.3	is more than before quarantine		28	I don’t pay attention to how healthy a food is. I consume/prepare foods that give me satisfaction	
16	I don’t consume those foods usually	During the lockdown period, your consumption of sweets:	17.3	I buy/consume more frozen/canned food than before quarantine	**During the quarantine period, the type of food that you prepare/consume:**
12	is less than before quarantine		62	I haven’t changed habits with respect to the type of food	
22	is the same as before quarantine				
50	is more than before quarantine		20.7	I buy/consume more fresh food than before quarantine (including fruit and vegetables)	

**Table 3:** Changes in weight and BMI after 1-month lockdown according to different characteristics.

Characteristics	Number	Weight Change (kg)	*p* *	BMI Change (kg/m^2^)	*p* *
*Gender*					
Male	34	+1.30		+0.46	
Female	116	+1.57	0.38	+0.62	0.75
*Age class (years)*					
≤50	76	+1.24		+0.47	
>50	74	+1.80	0.68	+0.70	0.48
*Living alone*					
No	76	+1.84		+0.71	
Yes	74	+1.17	0.50	+0.45	0.49
*Not working/retired*					
No	61	+1.64		+0.65	
Yes	89	+1.42	0.90	+0.54	0.79
*Active smokers*					
No	126	+1.50		+0.58	
Yes	24	+1.57	0.71	+0.59	0.78
*Secondary schools/graduation*					
No	57	+2.53		+0.98	
Yes	93	+0.89	0.013	+0.34	0.012
*Weight cycling*					
No	70	+1.14		+0.46	
Yes	80	+1.84	0.23	+0.69	0.27
*Night eating*					
No	132	+1.34		+0.51	
Yes	18	+2.75	0.11	+1.10	0.12
**During the lockdown period:**					
*Still working at workplace*					
No	127	+1.51		+0.58	
Yes	23	+1.50	0.61	+0.57	0.54
*Exercise levels*					
None or lower	119	+1.85		+0.70	
Usual or higher	31	+0.23	0.012	+0.13	0.011
Change in dietary habits **					
No/minimal changes	71	+0.86		+0.34	
Increased eating	60	+3.14		+1.20	
Decreased eating	19	−1.17	<0.001	−0.45	<0.001
*Conditions impacting on eating habits*					
*Boredom/solitude*					
No	96	+1.03		+0.40	
Yes	54	+2.37	0.012	+0.91	0.011
*Anxiety/depression*					
No	93	+0.49		+0.20	
Yes	57	+3.18	<0.001	+1.21	<0.001
*Food availability*					
No	121	+1.31		+0.51	
Yes	29	+2.37	0.16	+0.89	0.16
Consuming healthy foods **					
No	42	+3.70		+1.42	
Not changed	84	+1.08		+0.41	
Yes	24	−0.81	<0.001	−0.30	<0.001
Type of food **					
More frozen/canned foods	26	+2.21		+0.85	
Not changed	93	+1.22		+0.49	
More fresh foods	31	+1.80	0.08	+0.64	0.08
*Use of food delivery*					
No	130	+1.40		+0.53	
Yes	20	+2.25	0.33	+0.90	0.25
*Number of snacks*					
None or lower	59	+0.55		+0.20	
Usual or higher	91	+2.14	0.007	+0.83	0.005
*Cereal consumption*					
None or lower	28	−0.54		−0.19	
Usual or higher	122	+1.98	<0.001	+0.76	<0.001
*Protein consumption*					
None or lower	28	+1.42		+0.54	
Usual or higher	122	+1.53	0.99	+0.59	0.95
*Fruit/vegetable consumption*					
None or lower	28	+1.62		+0.63	
Usual or higher	122	+1.49	0.57	+0.57	0.58
*Sweet consumption*					
None or lower	42	−0.36		−0.14	
Usual or higher	108	+2.24	<0.001	+0.86	<0.001

* *p* by Mann–Whitney test; ** *p* by Kruskal–Wallis test.

**Table 4:** Multiple regression analyses of the variables associated with weight and BMI changes during the lockdown period.

Variable	Weight Change			BMI Change		
	*β*	95%CI	*p*	*β*	95%CI	*p*
**Secondary schools/graduation**	−1.15	−2.13, −0.17	0.022	−0.46	−0.83, −0.09	0.016
None/lower exercise	0.25	−0.97, 1.47	0.69	0.04	−0.41, 0.49	0.88
Boredom/solitude	0.004	−1.04, 1.04	0.99	−0.003	−0.39, 0.39	0.99
**Anxiety/depression**	1.61	0.53, 2.69	0.004	0.60	0.19, 1.01	0.004
Increased eating	0.78	−0.49, 2.05	0.23	0.29	−0.18, 0.76	0.24
**Not consuming healthy foods**	1.48	0.19, 2.77	0.026	0.59	0.10, 1.08	0.019
Usual/higher sweets	0.93	−0.29, 2.15	0.14	0.38	−0.07, 0.83	0.10
